# Judging a book by its older adult cover: age-related expectations and parental preference for children’s books

**DOI:** 10.1007/s12144-023-04298-6

**Published:** 2023-02-13

**Authors:** Jennifer A. Bellingtier, Lena-Emilia Schenker, Anna L. Weber

**Affiliations:** Department of Developmental Psychology, Friedrich Schiller University Jena, Jena, Germany

**Keywords:** Ageism, Children’s books, Vicarious contact, Stereotypes, Social learning

## Abstract

As they typically have limited direct contact, children’s attitudes towards older adults have more opportunity to be shaped by other social influences such as their parents and children’s literature. Children’s books have been noted for their tendency to portray older adults in stereotypical ways and their tendencies to underrepresent older adults. We investigated how the portrayal of older adults as major versus supporting characters, as well as parents’ age-related expectations were related to parental preference for children’s books. We designed 24 children’s book covers that depicted an older adult as a main character, a younger adult as a main character, or only children. One-hundred-seventy-five parents of children ages 0–12 rated their preference for the covers, their age-related expectations for the books’ stories, and their personal aging expectations. Parents preferred covers featuring only children, and this preference was stronger for parents with more positive personal aging expectations. Cover preference was further predicted by age-related story expectations. When parents expected a book to conform to older-age stereotypes, they liked that particular cover less. Controlling for parents’ age-related story expectations for each book resulted in near equal levels of preference for all types of book covers. Carefully designed children’s books could provide an opportunity to increased vicarious intergenerational contact. These finding suggests that parents’ interest in selecting these books for their children will be higher when they do not perceive the books to align with older-age stereotypes.

**Supplementary information:**

The online version contains supplementary material available at 10.1007/s12144-023-04298-6.

## Introduction

Negative views of older adults begin to form early in life with past research having documented negative associations in children as young as four years old (Bellingtier et al., 2021; Montepare & Zebrowitz, 2002). These negative views are known as ageism, which inaccurately describes older adults as a homogenous group primarily characterized by declining physical and mental abilities. The early development of ageism is especially disconcerting as these negative views have harmful consequences not only for the current population of older adults, but for the children themselves when they reach older age (Bellingtier & Neupert, 2018; Bergman, 2017; Levy et al., 2002a, b; Levy, 2009). Therefore, it is important to investigate possible pathways through which children’s aging attitudes may be shaped.

In modern western societies, children often lack frequent direct contact with older adults (Moorman et al., 2017; Seefeldt et al., 1977). Instead, their attitudes may have more opportunities to be influenced by the views of their parents (Degner & Dalege, 2013; Lineweaver et al., 2017) and the media (Bergman, 2017). In this study, we sought to investigate one possible pathway shaping children’s views of older adults: parental selection of children’s books. We examined parents’ preferences for children’s book covers that varied in the extent to which they featured older adults. We also examined if parents’ age-related expectations influenced their preferences.

### Children’s books and vicarious contact

Past research suggests that intergenerational contact can help to shape more positive views of older adults (e.g., Flamion et al., 2019; Teater & Chonody, 2017). Yet, opportunities for direct intergenerational contact may be limited due to age-segregation in local communities (Moorman et al., 2017), geographical distance from grandparents, or deliberate contact restrictions to protect older adults’ health in pandemic conditions (Frenkel-Yosef et al., 2020). Fortunately, past research suggests that positive contact effects can emerge through indirect forms of contact (Dovidio et al., 2011), such as vicarious contact. Vicarious contact occurs when individuals witness successful intergroup contact without directly experiencing it themselves (White et al., 2021; Wright et al., 1997). Children’s books can provide an opportunity to experience vicarious intergroup contact by portraying positive interactions between children and older adults. Furthermore, children’s books are often selected by their parents, important adult role models, and shared during close interpersonal encounters, such as bedtime, adding to their potential to shape children’s attitudes (Montepare & Zebrowitz, 2002).

Unfortunately, many studies highlight the negative stereotypical (Ansello, 1977; Avcı & Erhan, 2022; Barnum, 1977; Hollis-Sawyer & Cuevas, 2013; Robinson & Howatson-Jones, 2014; Sciplino et al., 2010) and one-dimensional characterizations (Ansello, 1977; Barnum, 1977; Crawford & Bhattacharya, 2014; Peterson & Eden, 1977; Robin, 1977) of older characters in children’s literature. Stereotypical or negative depictions of minority-group members in the media have been associated with more stereotypical attitudes and discrimination towards those groups. These connections have been found for media exposure and gender-stereotypical behaviors in children (Coyne et al., 2014, 2016; Seitz et al., 2020) and for media exposure and ageism in adults (Donlon et al., 2005; Haboush et al., 2012).

Furthermore, past research examining the portrayal of older adult characters in children’s literature has shown that older characters are underrepresented and rarely play major roles in children’s books. This was found to be the case for books from the *Children’s Catalog*, a compilation of prize-winning literature selected by educators and librarians (Barnum, 1977), Caldecott Medal award winners (Dellmann-Jenkins & Yang, 1997), *New York Times* and *Book Sense* bestsellers (Danowski & Robinson, 2012), and a more recent random sample of books (Hollis-Sawyer & Cuevas, 2013). Underrepresentation is one way personal attributes, such as age, become salient to children, and more salient attributes are more likely to become categories children use to form stereotypes (Bigler & Liben, 2007).

Thus, children’s books may not be fulfilling their potential as a source of positive vicarious intergenerational contact. The negative depiction and underrepresentation of older adults could reflect general societal biases against older adults as well as age-related biases on the part of authors, publishers, and booksellers (Cox & Young, 2020). It is also possible that those in the book industry assume that parents will be less interested in purchasing books featuring positive depictions of older adults. Here, we examined if parents do indeed have a preference for book covers that either exclude older adults or display them as only minor characters.

### Role of parents regarding the formation of stereotypes

Parents play a crucial role in shaping their children’s environment. Degner and Dalege’s (2013) large meta-analysis with data from over 45,000 parent-child dyads revealed that stereotypes held by parents correlate with those of their children. Similar results were found specifically for stereotypes about aging, which were shown to significantly correlate between fourth- and eighth-grade children and their parents (Lineweaver et al., 2017). Allport (1954) emphasizes the importance of parental influence on children’s development of intergroup prejudice through the creation of an atmosphere conducive to the development of similar stereotypical beliefs. Thus, parents’ own views and expectations regarding aging are likely to influence the social environment they create for their children. As young children usually do not purchase their own books, parents have discretion in the selection of children’s literature. Parents with ageist attitudes might prefer books that focus on children and younger adults over books featuring older adults. With books being an influential source of social learning (Strouse et al., 2018), parents could thus create an environment that fosters the development of ageist attitudes in their children. On the other hand, parents with positive aging expectations might present their children with books through which they can learn about and experience vicarious contact with older adults.

In the current study, we considered two types of age-related expectations. First, we examined parents’ personal aging expectations in regards to their perceptions that older adulthood is a time of declining physical and mental abilities (Sarkisian et al., 2005). When groups, such as older adults, are perceived more negatively, individuals tend to dissociate and distance themselves from these groups (Tajfel & Turner, 1986; Weiss & Kornadt, 2018). Therefore, we expected that when parents hold more negative expectations regarding aging they would, in turn, have lower preferences for children’s book covers featuring older adults. Second, we examined parents’ age-related expectations for the storylines of children’s books. If parents expect the stories contained within books that feature older adults on their covers to conform to age-related stereotypes (e.g., old-fashioned and boring) then we expected they would have lower preferences for these books (cf. Voss et al., 2018). In sum, we expected that parental preference for book covers featuring older adults would be lower when parents expected older adulthood in general, as well as books featuring older adults in particular, to conform to ageist stereotypes.

### The current study

Children’s books have the potential to be a positive source of vicarious intergenerational contact, yet past research indicates that many children’s books on the market either exclude older adults entirely or portray them in marginalized roles. To the best of our knowledge, the relationship between the inclusion of older adults in children’s books and preferences for these books has yet to be investigated. In the current study, we created fictitious children’s book covers that featured either an older or a younger adult as the primary character alongside a child, and asked parents to rate the book covers. We hypothesized that parents would prefer covers that feature younger, compared to older, adults (H1). Furthermore, we examined how parents’ age-related expectations would influence their preferences. We expected that parents with more negative expectations regarding aging in general (H2a) and with more age-stereotypical expectations of book covers featuring older adults (H2b) would have a stronger preference for covers featuring younger, as compared to older, adults.

## Method

### Participants

Participants included 187 parents or legal gaurdians of at least one child born in 2013 or later (i.e., 8 years old or younger). Parents ranged in age from 18 to 55 (*M* = 33.60, *SD* = 6.76), 52% identified as male, 47% identified as female, 1% non-binary, and about half held a university degree (53%). The majority had one (51%) or two (33%) children.

Sample size was determined using an online tool (jakewestfall.org/power/) for power analyses with crossed random effects (Westfall et al., 2014). We specified a counterbalanced design with 16 stimuli^1^, *d* = 0.50, variance around the participant and stimulus intercept at 0.2, variance around the stimulus and participant slope at 0.1, and residual variance at 0.4. This yielded a result of 154 participants. As we expected some participants would be excluded, we over-recruited to ensure the final sample size would be sufficient.

For data analysis, we excluded participants who indicated that they were very distracted in our attention check (*n* = 1) and those who took less than 12 min to complete the survey (*n* = 10). In addition, we excluded one older adult participant (age 71) as their judgements of covers featuring older adult characters would reflect assessments of an in-group member whereas the other participating parents would be assessing an out-group member. This left an analytic sample size of 175.

### Procedure

Recruitment occured online via Prolific Academic in June 2021. Respondents were compensated with 4.35£ (the equivalent of 5.91 USD). Before beginning the survey, all participants gave informed consent. They were then asked to think about one of their children whilst answering the survey items regarding children’s books. Parents were asked to select a child between the ages of 4 to 8 if possible, and when not to select their child closest to this age range. Parents indicated thinking about boys (51%), girls (49%), and one non-binary child with ages ranging from 4 months to 12 years old (*M* = 4.77, *SD* = 2.18). Parents then rated 24 book covers as described below, followed by a manipulation check. On average, the study took 22 min to complete. The procedure and all materials were approved by the Ethics Committee of the Friedrich Schiller University Jena.

### Materials

**Fig. 1:**
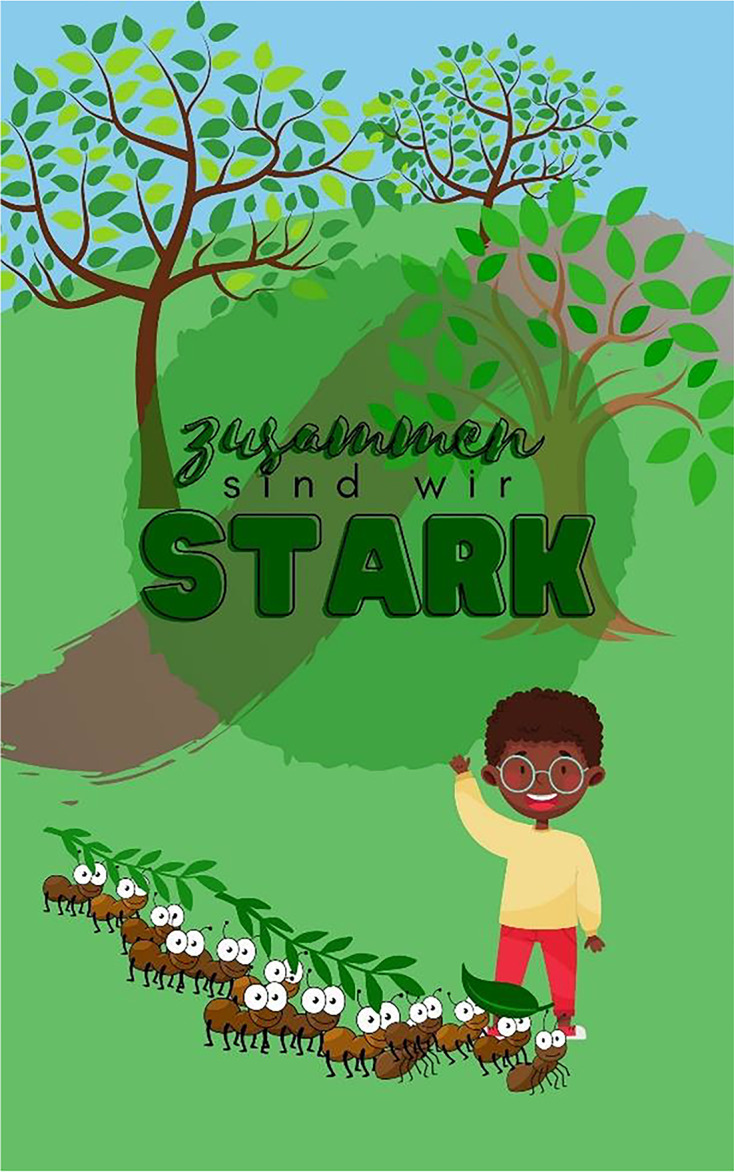
Example of a control cover. *Note*. The title translates to “*Stronger Together”*

Twenty-four children’s book covers were rated by each participant. Covers were designed for the study using the graphic-design program *Canva* with the intention to resemble real children’s books featuring male- and female-presenting characters with light and dark skin tones. All 24 covers featured a child embedded in settings inspired by real children’s books (e.g., everyday life, adventure, detective, and fantasy) with a title located above them. Eight control covers featured no additional people (see Fig. 1).

**Fig. 2:**
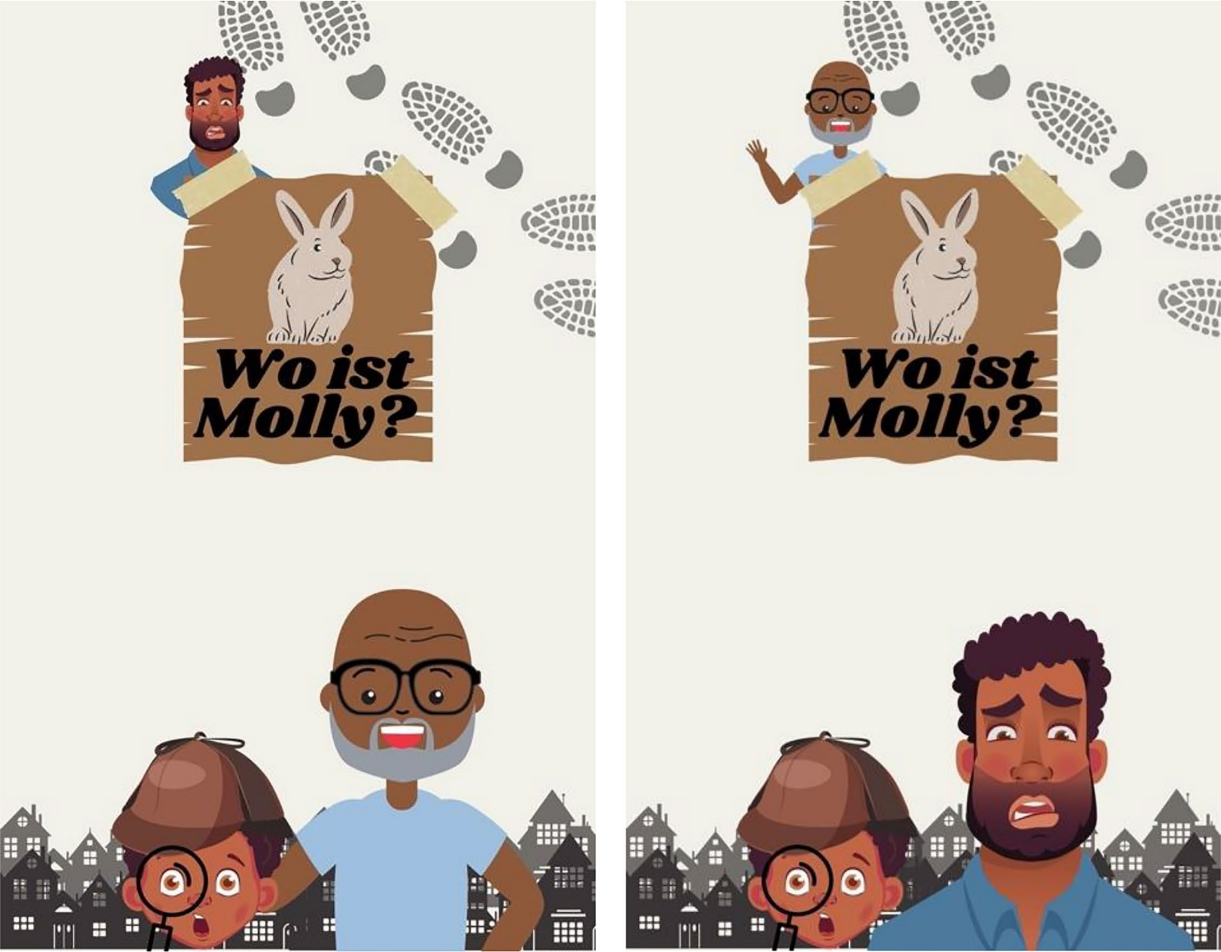
Examples of the experimental covers. *Note.* For each experimental cover, there was an older-version (left) and a younger-version (right). The title translates to “*Where is Molly?”*

The 16 experimental covers contained two additional people: an older and a younger adult both of whom matched the gender-appearance and skin-tone of the child. There were two versions of each experimental cover (see Fig. 2). In the older-version (OV), the older adult was featured in the foreground with the child, and the younger adult was in the background. In the younger-version (YV), the younger adult was featured in the foreground with the child, and the older adult was in the background. In all other ways the two versions of the experimental covers were identical. Participants rated eight of the experimental covers in their older-version and eight of the experimental covers in their younger-version.

All book covers were pre-tested with a sample of *N* = 24 undergraduates to confirm: (1) the child was perceived as 12 years old or younger, (2) the younger and older adults featured on each cover were perceived as at least 25 years apart in age with the younger adult perceived as at least 18 years old and the older adult as at least 55 years old, and (3) the child was perceived as a main character and the background adult was perceived as a supporting character. In addition, these requirements were further verified by the parent participants at the end of the current study. To not overburden these participants, three covers were randomly selected at the end of survey, and parents rated the perceived age and main-character status of the individuals on the covers. The parents perceived the characters as intended, both in terms of age (91% correctly rated the child’s age, 85% correctly rated the younger and older adult) and main-character status (87% rated the child as the main character, 91% rated the background adult as a supporting character).

### Measures

#### Ratings of children’s book covers

Each participant rated all eight control covers but only one version of each of the 16 experimental covers (8 older-version and 8 younger versions) for a total of 24 covers. The order and assignment of cover versions was randomized per participant with the caveat that participants rated an equal number of covers featuring older/younger, male/female, and lighter/darker-skinned characters.

##### Preference ratings

Parents’ liking of the books was measured with three items: “I like this book,” “My child would like this book,” and “I would buy this book if I were looking for a children’s book in a bookshop.” Responses were given on a 5-point Likert scale (1 = *strongly disagree* to 5 = *strongly agree*). Mean scores were created per book for each individual. Higher scores represent a higher preference for the books.

##### Age-related story expectations

To measure the participants’ age-related expectations regarding the storylines, parents responded to the statement “I think the story is…” followed by seven adjective pairs: *interesting* – *uninteresting*, *eventful* – *uneventful*, *exciting* – *dull*, *modern* – *traditional*, *progressive* – *old-fashioned*, *imaginative* – *boring*, *happy* – *sad*.

The adjectives were chosen based on three different sources. The first source was literature outlining prevailing stereotypes about older adults in the population (Mendonça et al., 2018). Parents may apply these stereotypes to the older adult characters featured on the book covers and expect the storyline to align with the stereotypes. The second source is literature outlining prevalent stereotypes in children’s literature (Crawford & Bhattacharya, 2014). Because parents usually read children’s books with their children, they are likely used to these stereotypical depictions of older adults in the stories. Thus, it might be expected that they expect new stories featuring older adults to be similar to the ones they know. The last source was the German Aging Semantic Differential scale (Gluth et al., 2010)^2^, a widely used scale for the assessment of age-related stereotypes in adults.

Five radio buttons were placed between each adjective pair and coded such that complete endorsement of the older-age-related adjectives (e.g., old-fashioned) was coded as 5 whereas complete endorsement of the younger-age-related adjectives (i.e., progressive) was coded as 1. Mean scores were created per book cover for each individual. Higher scores indicate story expectations that are more consistent with older age stereotypes.

#### Personal aging expectation

Parents’ expectations regarding aging in general were assessed with the German translation (Kessler et al., 2015) of the 12-item Expectations Regarding Aging scale (ERA-12; Sarkisian et al., 2005). Items, such as “It’s normal to be depressed when you are old,” were rated on a 4-point Likert scale ranging from 1 = *definitely false* to 4 = *definitely true*. After first reverse-scoring each item, scale scores were computed following the recommendations of Sarkisian et al. (2005). Scores ranged from 0 to 100 with higher scores indicating more positive expectations regarding aging (internal consistency, *α* = 0.84).

#### Analyses

Statistical analyses were conducted using SPSS Version 25. We adopted a multilevel modeling approach, as compared to ANOVA, so that random effects for both participants and stimuli could be included, which reduces the risk of Type I error and allows findings to generalize across other possible participants and stimuli (cf. Judd et al., 2017; Westfall et al., 2014). To address our first hypothesis that parents would prefer covers featuring younger rather than older adults, we began with a multilevel model with random effects for participants and stimuli.

1a $${\mathrm{Rating}}_{\mathrm{ijk}}={\mathrm\beta}_0+{\mathrm\beta}_1c_{ijk}+\alpha_i^P+\alpha_i^{P\times C}c_{ijk}+\alpha_j^S+\alpha_j^{S\times C}c_{ijk}+\varepsilon_{ijk}$$

In this equation, the book ratings of individual (i) for stimulus (j) in condition (k) are modeled by the overall mean rating (β_0_) and the conditional difference in ratings (β_1_c_*ijk*_). We initially modeled five random effects, which represented the variance around the intercept for participants (α_i_^P^) and for stimuli (α_j_^S^), around the conditional slope for participants (α_i_^P x C^c_*ijk*_) and for stimuli (α_j_^S x C^c_*ijk*_), and residual variance (ε_ijk_). There was no significant variance around the slope for participants nor for stimulus, and these random effects were removed in the interest of parsimony, which resulted in this final equation.

1b $${\mathrm{Rating}}_{\mathrm{ijk}}={\mathrm\beta}_0+{\mathrm\beta}_1c_{ijk}+\alpha_i^P+\alpha_j^S+\varepsilon_{ijk}$$

To address our second hypothesis that more negative age-related expectations would be associated with lower preference ratings for books featuring older adults, we modified Eq. 1b to further specify fixed effects for between-person differences in personal aging expectations.

2 $${\mathrm{Rating}}_{\mathrm{ijk}}={\mathrm\beta}_0+{\mathrm\beta}_1c_{ijk}+{\mathrm\beta}_2{\mathrm{PE}}_{\mathrm i}+{\mathrm\beta}_3c_{ijk}\times{\mathrm{PE}}_{\mathrm i}+\alpha_i^P+\alpha_j^S+\varepsilon_{ijk}$$

Here, β_2_PE_i_ represents the main effect of personal aging expectations on book ratings, and β_3_*c*_*ijk*_ x PE_i_ represents the cross-level interaction of personal aging expectations with condition. Personal aging expectation scores were grand-mean centered, thus the intercept reflects the average preference rating for an individual with sample-mean personal aging expectations.

Next, we modeled the association of parents’ age-related story expectations per book by modifying Eq. 1b to include the main effect of age-related story expectations (β_2_SE_ijk_) and the interaction of story expectations with condition (β_3_*c*_*ijk*_ x SE_ijk_).

3 $${\mathrm{Rating}}_{\mathrm{ijk}}={\mathrm\beta}_0+{\mathrm\beta}_1c_{ijk}+{\mathrm\beta}_2{\mathrm{SE}}_{\mathrm{ijk}}+{\mathrm\beta}_3c_{ijk}\times{\mathrm{SE}}_{\mathrm{ijk}}+\mathrm\alpha_{\mathrm i}^{\mathrm P}+\;\mathrm\alpha_{\mathrm i}^{\mathrm P\times\mathrm{SE}}{\mathrm{SE}}_{\mathrm{ijk}}+\mathrm\alpha_{\mathrm j}^{\mathrm S}+\mathrm\alpha_{\mathrm j}^{\mathrm S\times\mathrm{SE}}{\mathrm{SE}}_{\mathrm{ijk}}+{\mathrm\varepsilon}_{\mathrm{ijk}}$$

In addition to the random effects specified in Eq. 1b, we modeled two additional random effects: variance around the story expectation’s slope attributable to participants (α_i_^P x SE^SE_ijk_) and attributable to stimuli (α_j_^S x SE^SE_ijk_). Age-related story expectations were within-person centered, thus the estimates for story expectations reflect the change in book preferences when individuals indicate higher or lower age-related story expectations than their average rating across all book covers. Within-person centering controls for the possibility that some individuals might generally tend to perceive children’s stories as more or less age-stereotypical than other individuals.

## Results

Table 1 displays descriptive statistics for the study variables.

**Table 1:** Descriptive statistics for study variables

Variable	*M (SD)*	Range
Book preference		
Older versions	3.50 (0.70)	1.38–5.00
Younger versions	3.48 (0.68)	1.75–5.00
Control covers	3.85 (0.60)	2.04–5.00
Age-related story expectations		
Older versions	2.44 (0.59)	1.00–4.29
Younger versions	2.39 (0.57)	1.07–3.82
Control covers	2.14 (0.51)	1.00–3.54
Personal Aging Expectations	45.35 (18.33)	2.78–100.00

Greater book preference scores indicate stronger preference for the cover. Greater story expectation scores indicate the story is expected to more strongly conform to stereotypes about older adults. Higher values for personal aging expectations indicate more positive expectations regarding aging

### Parents’ book preferences

Multilevel model results for our first hypothesis did not indicate a greater preference for books featuring younger versus older adults on their covers, but a significantly greater preference for control covers in comparison to older version covers (see Table 2, column H1).

**Table 2:** Multilevel models for book preference ratings

Components	H1: Book Version Only		H2a: Personal Expectations^a^		H2b: Story Expectations^b^	
	Estimate	*95% CI*	Estimate	*95% CI*	Estimate	*95% CI*
Fixed effects						
Intercept	**3.50**	3.35, 3.64	**3.50**	3.35, 3.64	**3.61**	3.50, 3.73
Version Young	-0.01	-0.08, 0.06	-0.01	-0.03, 0.04	**-0.05**	-0.10, -0.01
Version Control	**0.36**	0.15, 0.56	**0.36**	0.15, 0.56	0.07	-0.06, 0.19
Expectations^c^			-0.001	-0.01, 0.004	**-0.91**	-0.99, -0.83
Expectations x Young			-0.001	-0.01, 0.003	-0.03	-0.10, 0.03
Expectations x Control			**0.01**	0.002, 0.01	-0.04	-0.16, 0.08
Random effects						
P Intercept	**0.33**	0.26, 0.41	**0.33**	0.26, 0.41	**0.35**	0.28, 0.43
P x E Slope					**0.05**	0.03, 0.07
S Intercept	**0.05**	0.02, 0.09	**0.05**	0.02, 0.09	**0.02**	0.01, 0.03
S x E Slope					**0.01**	0.01, 0.03
Residual Variance	**0.82**	0.78, 0.85	**0.81**	0.78, 0.85	**0.37**	0.35, 0.39

Older version covers are the reference version. P = participant; E = age-related story expectations; S = stimulus; CI = 95% confidence interval. Estimates significant at *p* < .05 are in bold

^a^Personal aging expectations are grand-mean centered in this model. ^b^Age-related story expectations are within-person centered in this model. ^c^Expectations refer to personal aging expectations in the H2a model and to age-related story expectations for individual books in model H2b

### Book preferences and personal aging expectations

Multilevel model results for Hypothesis 2a regarding personal aging expectations indicated that personal aging expectations moderated book preference ratings between older version and control covers (see Table 2, column H2a).

**Fig. 3:**
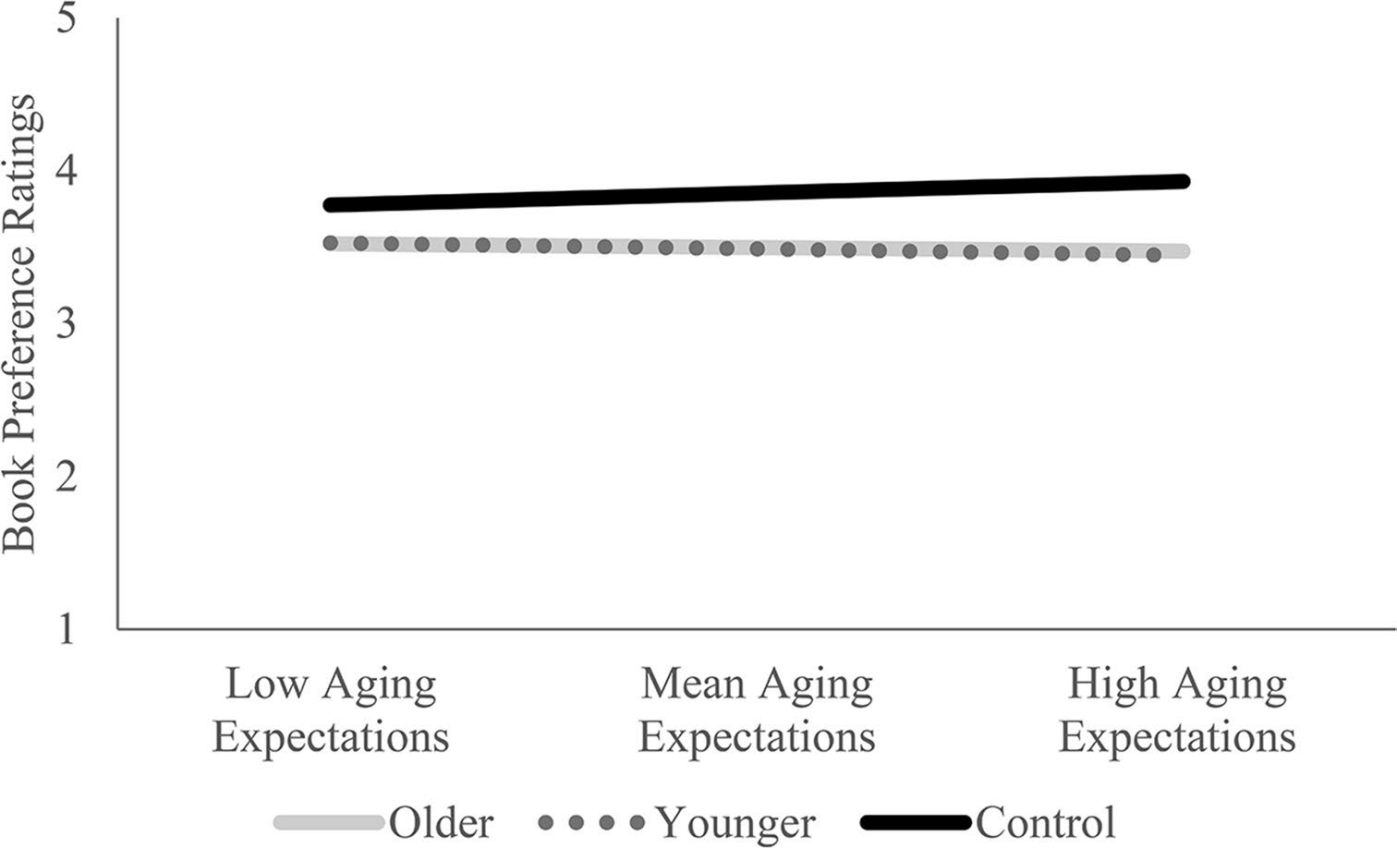
Book preference ratings for low, mean, and high levels of personal aging expectations. *Note*. Comparison of book preference ratings for low (-1SD), mean, and high (+ 1SD) personal aging expectations. Higher preference rating scores indicate a stronger preference for the book covers. Higher personal aging expectations scores indicate more positive expectations regarding personal aging

The interaction effect was examined by estimating preference ratings for the three types of covers at low, mean, and high levels of personal aging expectations, as shown in Fig. 3. The higher preference for control covers can be seen at all levels but is most pronounced for those with higher personal aging expectations.

### Book preferences and age-related story expectations

Hypothesis 2b was examined in regard to age-related story expectations. Multilevel model results indicated that when age-related story expectations were more consistent with older-age stereotypes, book preference ratings decreased (see Table 2, column H2b). The significant variance around the expectation slopes indicates that this effect varies by participants and for book stimuli. Age-related story expectations did not interact with book cover versions. However, including age-related story expectations in the model did result in a significant effect of cover version between younger and older versions with a greater preference for the older versions. This effect remained when the model was rerun removing the interaction term.

### Exploratory analyses for gender, skin tone, and parental age

We reran all models controlling for parent’s age, gender, education level, and number of children as well as their interaction with the predictor variables in each respective model. The pattern of findings remained the same, and none of these variables predicted cover preference nor interacted with any other predictor variables in any of the models.

We explored possible cover preference differences based on the gender presentation and skintone of the characters on the book covers. There was not a significant difference between male and female presenting covers, β_1_*c*_*ijk*_ = -0.13, *p* = .271, *M*_male_ = 3.55, *SD*_male_ = 1.14, *M*_female_ = 3.67, *SD*_female_ = 1.05. On the other hand, there was a significant difference between dark and light skintone covers, β_1_*c*_*ijk*_ = 0.24, *p* = .032, *M*_dark_ = 3.48, *SD*_dark_ = 1.14, *M*_light_ = 3.72, *SD*_light_ = 1.06. We reran all models controlling for characters’ skin tones. The pattern of age and age expectation findings reported above remain unchanged. These models are presented in Supplementary Table 1. There were no differences in age-related story expectations based on the gender presentation or skin tone of the characters.

## Discussion

Our examination of parental preferences for children’s books indicates that parents tend to prefer books without older adults as either main or supporting characters on their covers. However, these findings were qualified by parents’ age-related expectations. Unexpectedly, parents with more positive personal aging expectations had an even greater preference for book covers without older adults as compared to those featuring an older adult as a main character. In line with our hypothesis, when parents judged books’ stories to conform more strongly to age-related stereotypes, they had a lower preference for those book covers. Interestingly, when we included age-related story expectations in our model, we found a small preference for books with older adults featured as main characters on their covers, as opposed to younger adults. Furthermore, when covers do not suggest books contain age-stereotypical stories, parents expressed equal liking and willingness to purchase children’s books featuring older adults, as compared to only children, on their covers. Exposure to books featuring older adults would have the potential to provide children with an opportunity for positive vicarious intergenerational contact (Wong et al., 2022).

### Parents’ preference for book covers featuring children over older and younger adults

In our first model, we compared preference for book covers featuring older adults to those featuring either younger adults or only children without regard for aging expectations (see Table 2, Model H1). In this model, we found no evidence to support our first hypothesis that parents would prefer book covers that featured a child and younger adult as main characters versus a child and an older adult. Instead, our findings suggested that covers that include adults (both older and younger) are liked less than covers featuring only children. The greater preference for the children-only control covers could be due to their greater typicality for this genre of books. Bestselling lists (The New York Times, 2022) show that the large majority of children’s books do not depict any adults on the cover, and parents may have preferred the books that seemed more familiar (Piters & Stokmans, 2000).

The lack of difference between ratings for the books featuring older and younger adults on their covers may have been due to our conservative approach to designing the experimental books. These covers were identical in every way besides the placement of the older and younger adults, that is, all experimental covers included both adults. It is possible that any parental bias against books containing older adults applied equally to covers displaying the older adults as main or supporting characters (cf. Gordon, 2020). Even though the older adults were smaller background characters on the book covers featuring younger adults, their mere presence on the cover might have suggested these books contained older-age-stereotypical stories (cf. Avcı & Erhan, 2022). Indeed, the age-related story expectation were more stereotypical for both types of experimental covers in comparison to the children-only control covers. It is also possible that parents perceived the three characters to be part of a family with the older adults in the role of grandparents. Perceptions of “grandparents” often do not align with, and are typically more positive than those of “older adults” more generally (Bellingtier et al., 2022; Newsham et al., 2021; although cf. Henneberg, 2010). Thus, both versions of the experimental covers might have been perceived similarly and associated with grandparents. Future research using books that contain only depictions of younger or older adults is needed to disentangle the current findings.

### Effects of age-related expectations

The above findings were qualified by examining two types of age-related expectations. We first considered the role of parents’ personal age-related expectations, that is, their expectations about the aging process in general (see Table 2, Model H2a). Inclusion of these expectations did not alter the prior main effects, that is, covers featuring only children were still preferred to covers featuring older adults, which did not differ from covers featuring younger adults. However, there was an interaction effect indicating that parents with more positive personal age-related expectations had the strongest preference for the children-only control covers over the experimental covers including older adults. This finding was contrary to our expectations and should be interpreted with caution. One possibility is that these parents with more positive personal age-related expectations are aware of the tendency for books containing older adult characters to contain stereotypical and negative portrayals of aging (e.g., Avcı & Erhan, 2022; Hollis-Sawyer & Cuevas, 2013; Robinson & Howatson-Jones, 2014) from which they wish to shield their children. For example, a recent bestselling book featuring an older adult on the cover begins with the line, “Another year, and I still don’t like old people. Their walker shuffle, their unreasonable impatience, their endless complaints, their tea and cookies, their bellyaching,” (Groen, 2014). Parents who want to ensure their children are not exposed to negative vicarious contact with older adult via books may then be especially likely to prefer book covers featuring only children (cf. Schemer & Meltzer, 2020), especially when they are only able to view the cover to make their decision.

We next considered the role of age-related story expectations for each individual book cover. In this model (see Table 2, Model H2b), we found that expectations for the particular book cover were the best predictor of parents’ book preferences such that a 1-point increase in age-related story expectations was associated with a 0.91-point decrease in book preference. When these book-specific expectations were included, the preference for children-only control covers relative to older versions disappeared. This would suggest that at least part of the difference in preference seen in the prior models can be attributed to differences in age-related story expectation. The comparison to younger cover versions was also significant in this final model; this indicates that once age-related story expectations have been considered, there is a very slight preference for covers featuring older versus younger adults. Overall, considering the age-related story expectations resulted in a relative similarity between the ratings for all three types of covers. The effect for age-related story expectations is likely greater than the effect of personal aging expectations as it was assessed at the same level (i.e., per individual book) as the outcome book preferences versus personal aging expectations that were measured at the individual level. In addition, the significant random effects suggest that books vary in the extent to which they activate older-age-related stereotypes regardless of the prominence of older adults on the covers. Thus, what seems to be most important for parents’ preferences is that the book’s story does not appear to be conforming to older-age stereotypes, which tend to be negative (e.g., Ansello, 1977; Hollis-Sawyer & Cuevas, 2013; Sciplino et al., 2010) and one-dimensional (Barnum, 1977; Crawford & Bhattacharya, 2014; Peterson & Eden, 1977; Robin, 1977). Although, we caution that parents may not be consciously considering age-stereotypes when contemplating their book preferences. The activation of age-related stereotypes may happen implicitly (de Paula Couto & Wentura, 2017; Kornadt et al., 2016).

These findings could have important implications for the reduction of ageism. Because many children have limited direct contact with older adults (Hagestad & Uhlenberg, 2006; Seefeldt et al., 1977), an alternative direction for increasing contact is through vicarious contact via books, which has proven beneficial in other realms of intergroup contact (Vezzali et al., 2014; Wong et al., 2022). Our findings suggest that those looking to increase contact with older adults via books should be especially mindful to select books that do not appear to conform to negative aging stereotypes. Selecting books that portray older adults with balanced and nuanced depictions could be a promising way of increasing children’s contact with older adults. Vicarious contact is particularly relevant in times of the global COVID-19 pandemic, during which the risk of children developing ageist stereotypes is high due to increased ageism and age-segregation (Lichtenstein, 2021).

### Strengths, limitations, and future directions

This study provides the first evidence regarding parents’ preferences for children’s book covers featuring older adults. Understanding the factors that make it more likely for parents to like and purchase these books is a necessary first step towards increasing positive vicarious intergenerational contact via books. By using a new approach and designing our own book covers, it was possible to experimentally manipulate the age of the characters on the cover while controlling for other possibly confounding aspects. Nevertheless, some limitations should be noted. First, our choice of design elements was limited to those available in the Canva design software. Subtle differences in the quality of the graphics available to represent children, younger adults, and older adults may exist and could have influenced our findings. Second, differences in book preferences related to character age were small in nature (i.e., -0.05 to 0.36 on a 5-point scale), although the effect of age-related story expectations was closer to one scale point (i.e., -0.91). Future work is needed to examine the practical implications of these differences. Third, as mentioned earlier, all experimental covers included both an older and younger adult. We had expected that manipulating the prominence of the older adult would result in differential preference ratings. There are multiple reasons why a difference may not have been found (e.g., the presence of any older adult reduces ratings compared to children-only covers, the presence of a younger adult suggests a family grouping with older adults perceived more positively as grandparents than they would be on their own, or a true lack of difference in preference for older and younger adults). Future work with different types of covers (e.g., comparing covers with only an older adult and child to those with only a younger adult and child) is needed to further understand this finding. It is also necessary for future work to further examine the relationship of aging expectations with book preferences. For example, future research could ask parents for their expectations for the depiction of each character in the story, as opposed to asking about the storyline in general as we did in this study. Lastly, there is a need for research linking vicarious contact with older adults, such as contact through books, to children’s attitudes towards them. Although this link has been established in adult samples (Donlon et al., 2005; Haboush et al., 2012) and for other types of intergroup attitudes in children (e.g., Coyne et al., 2014, 2016; Seitz et al., 2020), it has yet to be examined between children and older adults.

In conclusion, this study advances the understanding of social factors that can contribute to the formation of age-related attitudes in children. Our findings suggest that parents are more open to exposing their children to vicarious intergenerational contact via books when they perceive book covers to contain stories that do not conform to age-related stereotypes. Although book covers containing any adults were preferred less than books with only children, we did not find any evidence that parents’ preferences were lower when older adults were featured in a major versus supporting role on the covers. Thus, children’s books could potentially aid in the reduction of ageist stereotypes in children if their covers are designed in a way that does not activate older-age stereotypes in parents. Positive vicarious intergenerational contact presents an opportunity for shaping more positive and balanced views of aging in children.

## Supplementary information

Below is the link to the electronic supplementary material.

ESM 1 (DOCX 17.2 KB)

## Data Availability

The dataset generated during and analyzed during the current study are available in the OSF repository for this study, https://osf.io/u5kzx/?view_only=e98eb4b8adeb4b7c99994066e8068751.

## Footnotes

1 Power analysis was based on the comparison of any two conditions, each with 8 book covers (i.e., 16 cover ratings per person).

2 The adjective pairs that were adopted from the German Aging Semantic Differential scale are: *exciting* – *dull, progressive* – *old-fashioned*, and *happy* – *sad*.
