# Supplementary material for: Judging a book by its older adult cover: age-related expectations and parental preference for children’s books
Source: Curr Psychol. 2023 Feb 13:1–13. Online ahead of print. doi: 10.1007/s12144-023-04298-6 (PMC9923672; doi:10.1007/s12144-023-04298-6)
Supplement: Supplementary file 1 — (DOCX 17.2 KB) [file 12144_2023_4298_MOESM1_ESM.docx]

**Supplementary Table 1**

*Multilevel Models for Book Preference Ratings Including Skin Tone*

| Components | H1: Book Version Only | |  | H2a: Personal Expectations | |  | H2b: Story Expectations | |
| --- | --- | --- | --- | --- | --- | --- | --- | --- |
|  | Estimate | *95% CI* |  | Estimate | *95% CI* |  | Estimate | *95% CI* |
| Fixed Effects |  |  |  |  |  |  |  |  |
| Intercept | **3.39** | 3.23, 3.56 |  | **3.39** | 3.23, 3.56 |  | **3.59** | 3.46, 3.71 |
| Version Young | -0.01 | -0.08, 0.06 |  | -0.01 | -0.08, 0.06 |  | **-0.05** | -0.10, -0.01 |
| Version Control | **0.33** | 0.14, 0.52 |  | **0.33** | 0.14, 0.52 |  | 0.06 | -0.07, 0.18 |
| Expectations |  |  |  | -0.001 | -0.01, 0.004 |  | **-0.91** | -0.99, -0.83 |
| Expectations x Young |  |  |  | -0.001 | -0.01, 0.003 |  | -0.03 | -0.10, 0.03 |
| Expectations x Control |  |  |  | **0.01** | 0.002, 0.01 |  | -0.04 | -0.17, 0.08 |
| Skin Tone (light) | **0.20** | 0.03, 0.38 |  | **0.20** | 0.03, 0.38 |  | 0.05 |  |
|  |  |  |  |  |  |  |  |  |
| Random Effects |  |  |  |  |  |  |  |  |
| P Intercept | **0.33** | 0.26, 0.41 |  | **0.33** | 0.26, 0.42 |  | **0.35** | 0.28, 0.43 |
| P x E Slope |  |  |  |  |  |  | **0.05** | 0.03, 0.07 |
| S Intercept | **0.04** | 0.02, 0.07 |  | **0.04** | 0.02, 0.07 |  | **0.02** | 0.01, 0.03 |
| S x E Slope |  |  |  |  |  |  | **0.01** | 0.01, 0.03 |
|  |  |  |  |  |  |  |  |  |
| Residual Variance | **0.82** | 0.78, 0.85 |  | **0.81** | 0.78, 0.85 |  | **0.37** | 0.35, 0.39 |

*Note.* Older version covers are the reference version. Darker skin tones are the reference version. P = participant; E = age-related story expectations; S = stimulus; CI = 95% confidence interval. Estimates significant at *p* < .05 are in bold.
